# Protective Effects of Ferulic Acid against Heat Stress-Induced Intestinal Epithelial Barrier Dysfunction *In Vitro* and *In Vivo*

**DOI:** 10.1371/journal.pone.0145236

**Published:** 2016-02-19

**Authors:** Shasha He, Fenghua Liu, Lei Xu, Peng Yin, Deyin Li, Chen Mei, Linshu Jiang, Yunfei Ma, Jianqin Xu

**Affiliations:** 1 CAU-BUA TCVM Teaching and Researching Team, College of Veterinary Medicine, China Agricultural University (CAU), Beijing, P. R. China; 2 Beijing Key Laboratory of Dairy Cow Nutrition, College of Animal Science and Technology, Beijing University of Agriculture (BUA), Beijing, P. R. China; Indiana University School of Medicine, UNITED STATES

## Abstract

Heat stress is important in the pathogenesis of intestinal epithelial barrier dysfunction. Ferulic acid (FA), a phenolic acid widely found in fruits and vegetables, can scavenge free radicals and activate cell stress responses. This study is aimed at investigating protective effects of FA on heat stress-induced dysfunction of the intestinal epithelial barrier *in vitro* and *in vivo*. Intestinal epithelial (IEC-6) cells were pretreated with FA for 4 h and then exposed to heat stress. Heat stress caused decreased transepithelial electrical resistance (TER) and increased permeability to 4-kDa fluorescein isothiocyanate (FITC)-dextran (FD4). Both effects were inhibited by FA in a dose-dependent manner. FA significantly attenuated the decrease in occludin, ZO-1 and E-cadherin expression observed with heat stress. The distortion and redistribution of occludin, ZO-1 and E-cadherin proteins were also effectively prevented by FA pretreatment. Moreover, heat stress diminished electron-dense material detected in tight junctions (TJs), an effect also alleviated by FA in a dose-dependent manner. In an *in vivo* heat stress model, FA (50 mg/kg) was administered to male Sprague–Dawley rats for 7 consecutive days prior to exposure to heat stress. FA pretreatment significantly attenuated the effects of heat stress on the small intestine, including the increased FD4 permeability, disrupted tight junctions and microvilli structure, and reduced occludin, ZO-1 and E-cadherin expression. Taken together, our results demonstrate that FA pretreatment is potentially protective against heat stress-induced intestinal epithelial barrier dysfunction.

## Introduction

The intestinal epithelium forms a selective permeability barrier allowing absorption of water and nutrients while preventing access of luminal pathogens and antigens from the luminal environment into mucosal tissues and the circulatory system [1,2]. However, the selective permeable barrier is frequently disrupted by stress and a number of studies have demonstrated that heat stress can impair intestinal barrier function by damaging intestinal epithelium and increasing intestinal permeability [3–5]. As a consequence, pathogens and endotoxins enter the circulation, resulting in endotoxemia, inflammatory bowel disease (IBD) and organ dysfunction [6–8]. Therefore, protecting the integrity of the intestinal barrier is an important goal for treating heat stress.

The formation and effectiveness of the epithelial barrier depend on the junctional complexes, including tight junctions (TJs), adherence junctions (AJs) and desmosomes [1,9]. TJs, the most apical components of the junctional complexes, are composed of multiple proteins, including transmembrane proteins occludin, claudins, and peripheral membrane proteins zonula occludens-1 (ZO-1) and ZO-2 [10–12]. The proteins occludin and ZO-1 have been shown to play important roles in maintenance of TJ structure and epithelial barrier function [13]. E-cadherin, α-catenin 1, β-catenin, catenin δ1 and F-actin interact to form the adherens junction [1]. E-cadherin protein is the major constituent of adherens junctions, which play a key role in intestinal homeostasis and maintenance of the epithelial line of defense [14].

Ferulic acid (FA) belongs to the family of phenolic acids and is very abundant in fruits and vegetables [15]. It has potent antioxidant activity, scavenging free radicals and inhibiting lipid peroxidation [16,17]. Recently, ferulate was shown to protect the epithelial barrier by suppressing both an increase in epithelial permeability and a decrease in expression of the TJ proteins occludin and ZO-1 in Caco-2 cells [18]. However, no research has described protective effects of FA against heat stress-induced intestinal epithelial barrier dysfunction in IEC-6 cells and *in vivo*. Therefore, the goal of the present study was to investigate the ability of FA to protect IEC-6 cell monolayers against heat stress-induced epithelial barrier dysfunction. Additionally, we designed an *in vivo* study to assess the protection effect of FA against heat stress-induced intestinal epithelial barrier dysfunction.

## Materials and Methods

### Reagents

FA (purity >99%) was purchased from the National Institutes for Food and Drug Control (Beijing, China). Dulbecco’s Modified Eagle medium (DMEM), fetal bovine serum (FBS), antibiotic–antimycotic, and TRIzol^®^ reagent were purchased from Gibco (Grand Island, NY, USA). 3-(4,5-Dimethylth-iazol-2-yl)-2,5 diphenyl tetrazolium bromide (MTT) was from Sigma-Aldrich (St. Louis, MO, USA).

### Cell culture

IEC-6 intestinal epithelial cells were obtained from the Cell Resource Center (Beijing, China). Cells were cultured in DMEM medium supplemented with 10% fetal bovine serum, 100 U/ml antibiotics (penicillin and streptomycin) and 0.01 mg/ml insulin at 37°C in a humidified incubator with 5% CO_2_.

### Cytotoxicity assay

The MTT assay was used to determine effects of FA on IEC-6 cell viability. Briefly, cells were seeded at 1 × 10^4^ cells per well onto 96-well plates and allowed to grow to 90% confluence in complete medium. Cells were then washed twice with phosphate-buffered saline (PBS) and serum-starved for 2 h prior to incubation with FA (1, 5, 10, 20, 50, 100, 200, or 500 μM) for 48 h. Medium was then removed and 10 μl MTT solution (5mg/mL) was added to each well and incubated at 37°C for 4 h. Supernatants were removed, and dimethyl sulfoxide (Amerco, Solon, OH, USA) was added to each well. The solubilized formazan product was analyzed with a multi-detection microplate reader (Synergy HT, BioTek, Winooski, VT, USA) at a fixed absorption wavelength of 490 nm and reference wavelength of 630 nm.

### Measurement of TER

IEC-6 cells were seeded at 2.0 ×10^6^ cells per well on polycarbonate membranes in transwell inserts (6.5-mm diameter, 0.4-μm pore size; Corning, Cambridge, MA, USA) and monitored daily for 2 weeks by measuring TER. When TER values were consistently above 300 Ω/cm^2^, the experiments were performed. For groups receiving heat stress treatment, TER measurements were recorded prior to and after heat stress. For groups receiving FA, TER measurements were recorded prior to pretreatment with FA (0, 5, 10 or 20 μM) for 4 h and after heat stress. TER of the filter-grown IEC-6 intestinal monolayers was measured with a Millicell-ERS (electrical resistance system; Millipore, Bedford, MA, USA). TER was expressed in Ω/cm^2^, calculated by dividing the measured resistance by the surface area of the monolayer (0.33 cm^2^ for 6.5-mm wells). The resistance of the polycarbonate membranes in transwell inserts (approximately 30 Ω/cm^2^) was subtracted from all readings before calculating TER. Changes in TER under experimental conditions were expressed as a percentage of the corresponding basal values.

### Intestinal epithelial paracellular permeability assay

Intestinal epithelial paracellular permeability across cell monolayers was determined by measuring the flux of FITC-labeled dextran of molecular mass 4 KDa (FD4, Sigma-Aldrich). IEC-6 cell monolayers were pretreated with FA (0, 5, 10 or 20 μM) for 4 h and then exposed to heat stress for 6h. FD4 (1mg/ml) was added to medium in the apical chamber of transwells. After 2 h incubation, medium was collected from the bottom chamber and FITC was measured in a fluorometer (excitation, 492 nm; emission, 520 nm; BioTek).

### Western blot analysis

Proteins from IEC-6 cells or rat intestine samples were extracted using a total protein extraction kit (Biochain, Hayward, CA, USA) and quantified using the BCA protein assay kit (Pierce, Rockford, IL, USA). Proteins separated by SDS-PAGE were transferred to nitrocellulose membrane (Pierce). Membranes were blocked with Odyssey blocking buffer (LI-COR, Lincoln, NE) for 2 h at room temperature (RT). The blocked membranes were subsequently incubated with antibodies against occludin (Invitrogen, Carlsbad, CA, USA), ZO-1 (Santa Cruz Biotechnology, Santa Cruz, CA, USA), E-cadherin and β-actin (Cell Signaling Technology, Danvers, MA, USA) overnight at 4°C. The membranes were then washed and incubated with the secondary anti-rabbit and anti-mouse IgG antibodies (Santa Cruz Biotechnology) at a dilution of 1:15,000 for 1 h at RT. The protein bands were then scanned in the 800 nm channel of the Odyssey infrared imaging system (LI-COR Biosciences) and quantified with Odyssey software version 3.0 (LI-COR Biosciences).

### Immunofluorescence staining

IEC-6 monolayers were grown on coverslips to 100% confluence then treated with various experimental conditions, as indicated in Results. At the end of the experimental period, IEC-6 monolayers were washed twice in PBS and fixed with 4% paraformaldehyde for 10 min at RT. Then cells were permeabilized with 0.2% Triton X-100 in PBS at 4°C for 10 min, followed by blocking in 3% BSA for 1 h at RT. Cells were incubated overnight at 4°C with primary antibodies against ZO-1, occludin or E-cadherin, each at a 1:50 dilution. After washing with PBS, coverslips were incubated with Alexa Fluor 488 goat anti-rabbit and goat anti-mouse IgG secondary antibodies (Life Technologies, Molecular Probes) at a dilution of 1:400 for 40 min at RT in the dark. Cells were also incubated with DAPI (Beyotime, Jiangsu, China) for 5 min to stain nuclei. After washing three times with PBS, cells were mounted with Prolong Antifade medium (Solarbio, Beijing, China). Stained cell monolayers were examined with a fluorescence microscope (OLYMPUS IX71, Tokyo, Japan).

### Transmission electron microscopy (TEM)

IEC-6 cells were collected and centrifuged at 3000g for 15 min. cells were fixed in overnight with 4% glutaraldehyde and post-fixed in cold 1% osmium tetroxide. Subsequently, cells were dehydrated in a graded acetone series and embedded in epoxy resin. Ultra-thin sections were stained with saturated uranyl acetate in 50% ethanol and lead citrate and then examined with a JEM-1230 transmission electron microscope (JEOL, Tokyo, Japan).

### Animals and Ethics statement

Male Sprague-Dawley rats weighing 200 ± 20 g were obtained from Beijing Vital River Laboratory, Animal Technology Co., Beijing, China. All procedures performed on the animals were approved by the Animal Care and Use Committee of China Agricultural University (permit number: CAU20150806-2). The rats were housed at a constant temperature of 25°C, 60% relative humidity with a 12/12 h light-dark cycle and were allowed water *ad libitum*.

### Experimental design

Twenty-four rats were randomly divided into three groups (n = 8) and were orally administered various pretreatments for 7 consecutive days and otherwise treated, as follows: (A) control pretreated with 0.9% normal saline; (B) heat stress (HS) group, pretreated with 0.9% normal saline then exposed to 40°C and 60% relative humidity from 11:00 am to 1:00 pm daily for three consecutive days [19]; (C) FA+ HS group, pretreated with FA (50 mg/kg) and then exposed to 40°C and 60% relative humidity from 11:00 am to 1:00 pm daily for three consecutive days.

### Sample preparation

After the final 2 h heat treatment, the rats were anesthetized, and a midline laparotomy was performed. Plasma was collected by centrifuging the blood at 10,000g for 10 minutes at − 4°C for intestinal epithelial permeability analysis. The small intestine samples from each group were harvested for transmission electron microscopy and western blot analysis.

### Intestinal epithelial permeability analysis

An *in vivo* intestinal permeability assay was performed to assess intestinal barrier function as previously described [20]. Briefly, 30 min before sacrifice, rats were anesthetized with inhaled isoflurane. A midline laparotomy was performed and a 10-cm segment of the distal jejunum was isolated between silk ties. A solution of 1.0 mL phosphate-buffered saline (PBS, pH 7.2) containing 25 mg FD4 (Sigma, St Louis, MO, USA) was injected into the lumen of the isolated segment of the isolated jejunum. The bowel was then returned to the abdominal cavity and the abdomen was closed. Rats were maintained under light general anesthesia for 30 min, at which time systemic blood was collected. Plasma was collected by centrifuging the blood at 10,000g for 10 minutes at − 4°C. Plasma fluorescence was measured in a fluorescence spectrophotometer (Synergy HT, Biotek, Winooski, Vermont, USA). A standard curve for the assay was obtained through serial dilution of FITC-Dextran in rat plasma.

### Transmission electron microscopy (TEM)

The jejunum were fixed in overnight with 4% glutaraldehyde and post-fixed in cold 1% osmium tetroxide. Subsequently, jejunum were dehydrated in a graded acetone series and embedded in epoxy resin. Ultra-thin sections were stained with saturated uranyl acetate in 50% ethanol and lead citrate and then examined with a JEM-1230 transmission electron microscope (JEOL, Tokyo, Japan).

### Statistical Analysis

Statistical analysis was performed using the GraphPad Prism 5 program (GraphPad, La Jolla, CA, USA). All measurements in this study are represented as means ± SEM. Data were analyzed using Student’s t test when appropriate, or by a one-way analysis of variance (ANOVA) and Tukey’s multiple comparison tests were applied when comparing more than three means. Results were considered significant at P< 0.05.

## Results

### Heat stress-induced intestinal epithelial barrier dysfunction

**Fig 1:**
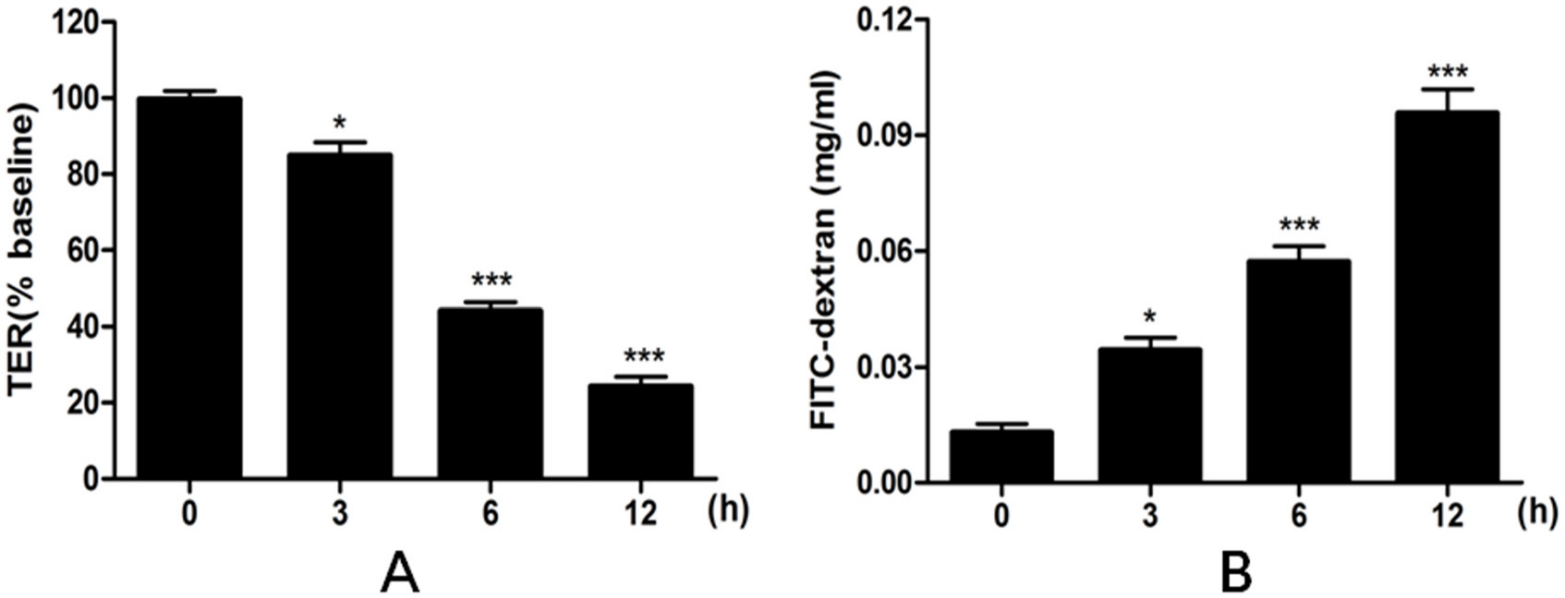
Effects of heat stress on TER and on FD4 permeability in IEC-6 cell monolayers. IEC-6 cells on Transwell membranes were exposed at 42°C for 0, 3, 6 or 12 h. (A) TER values were monitored across cell monolayers at the indicated times. (B) Permeability of FD4 across the cell monolayer was measured. Data are presented as means ± SEM from three independent experiments and differences among mean values were assessed by one-way ANOVA. p<0.05 and *** p<0.001 compared with the control group.

The effects of heat stress on epithelial barrier integrity and paracellular permeability were determined by measuring TER and the flux of FD4. Exposure to heat stress for up to 12 h produced a time-dependent decrease in TER, statistically significant by 6 h (p<0.001) (Fig 1A). Similarly, heat stress was associated with a time-dependent increase in IEC-6 paracellular permeability to FD4 (Fig 1B). The FD4 flux from apical to basolateral chamber was significantly increased by 6 h of heat stress (p<0.001). Therefore, exposure to heat stress for 6 h was selected as the condition for subsequent experiments.

### Heat stress regulated the expression of occludin, ZO-1 and E-cadherin

**Fig 2:**
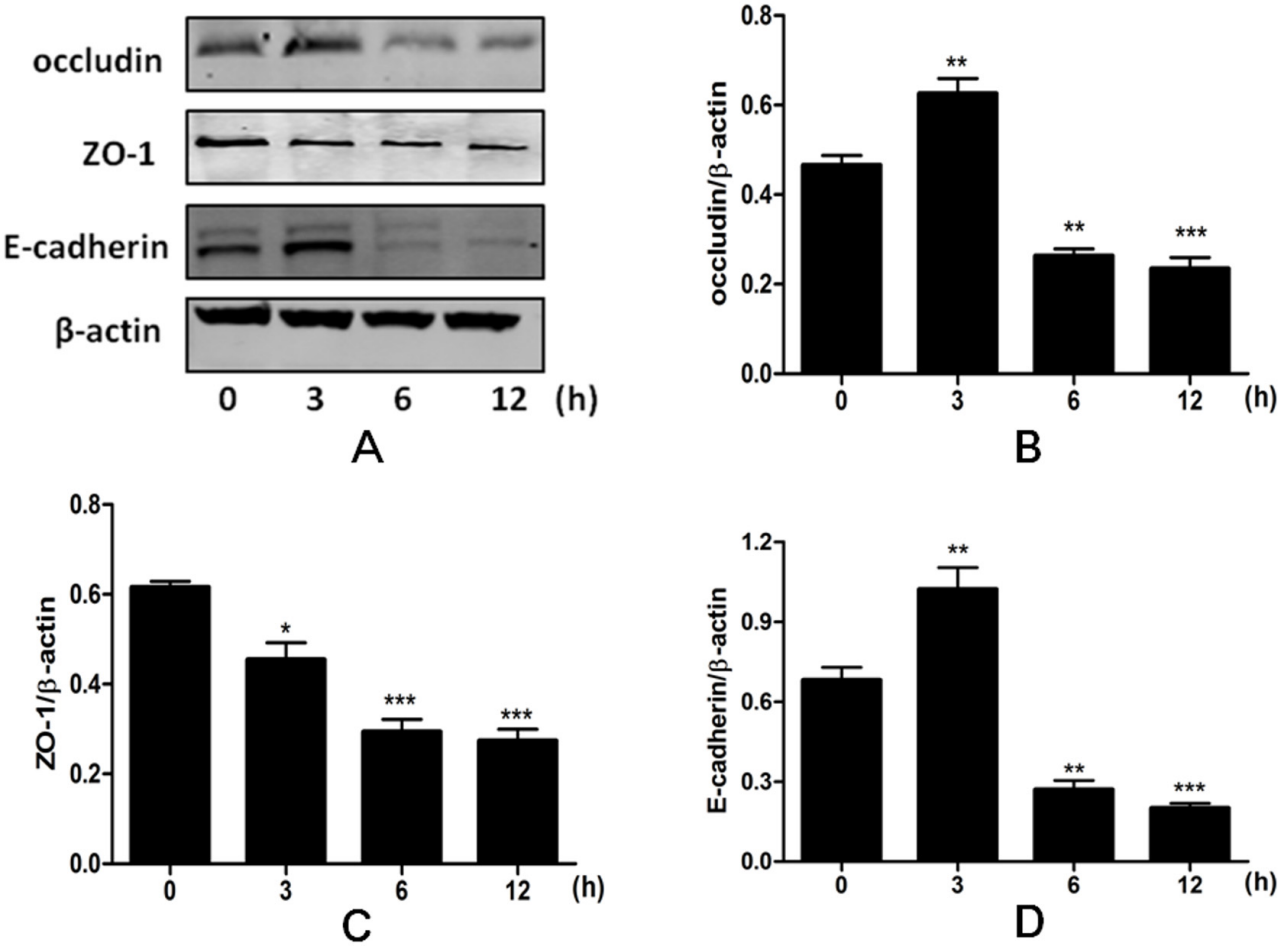
Time-dependent effects of heat stress on expression of occludin, ZO-1 and E-cadherin in IEC-6 cells. Cells were exposed to 42°C temperature for 0, 3, 6 or 12 h and total proteins harvested for western blotting assays. Data are presented as means ± SEM from three independent experiments and differences among mean values were assessed by one-way ANOVA. * p<0.05, ** p<0.01 and *** p<0.001 compared with the control group.

Because epithelial junctional complexes regulate paracellular permeability, we analyzed expression of three representative junctional complexes proteins, namely, occludin, ZO-1 and E-cadherin, by western blotting. Heat stress significantly decreased ZO-1 expression in a time-dependent manner (p<0.001). Expression of occludin and E-cadherin increased from 0 to 3 h (p<0.01), but was significantly decreased by 6 h after heat stress (p<0.01) (Fig 2).

### FA protected against heat stress-induced intestinal epithelial barrier dysfunction

**Fig 3:**
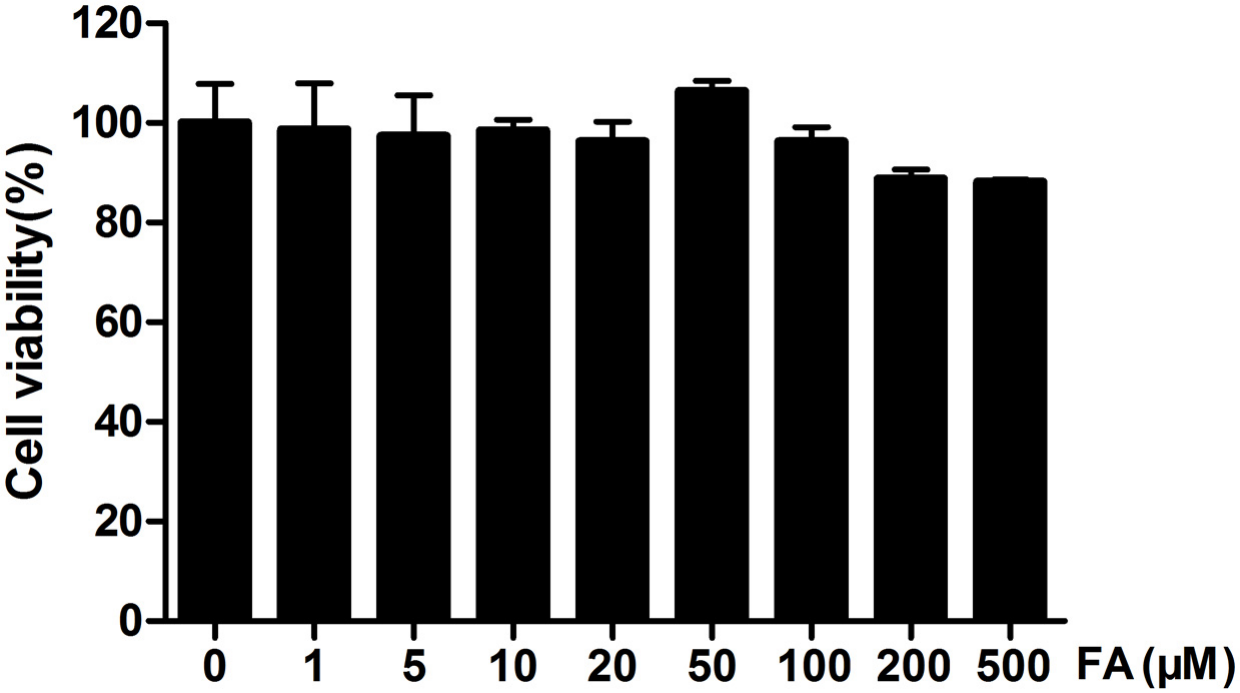
Cytotoxicity of FA on IEC-6 cells. Effects of FA on viability of IEC-6 cells was measured using the MTT assay. Cells were incubated with FA concentrations ranging from 0 to 500 μM for 48 h. Data are presented as means ± SEM from three independent experiments and differences among mean values were assessed by one-way ANOVA.

To exclude the possibility that FA had toxic effects under these experimental conditions, we conducted a cell viability test using the MTT assay. In IEC-6 cells incubated with various concentrations (0–500 μM) of FA for 48 h, FA had no cytotoxic effects (Fig 3). This finding justified our choice of 5, 10, 20 μM FA for all subsequent experiments.

**Fig 4:**
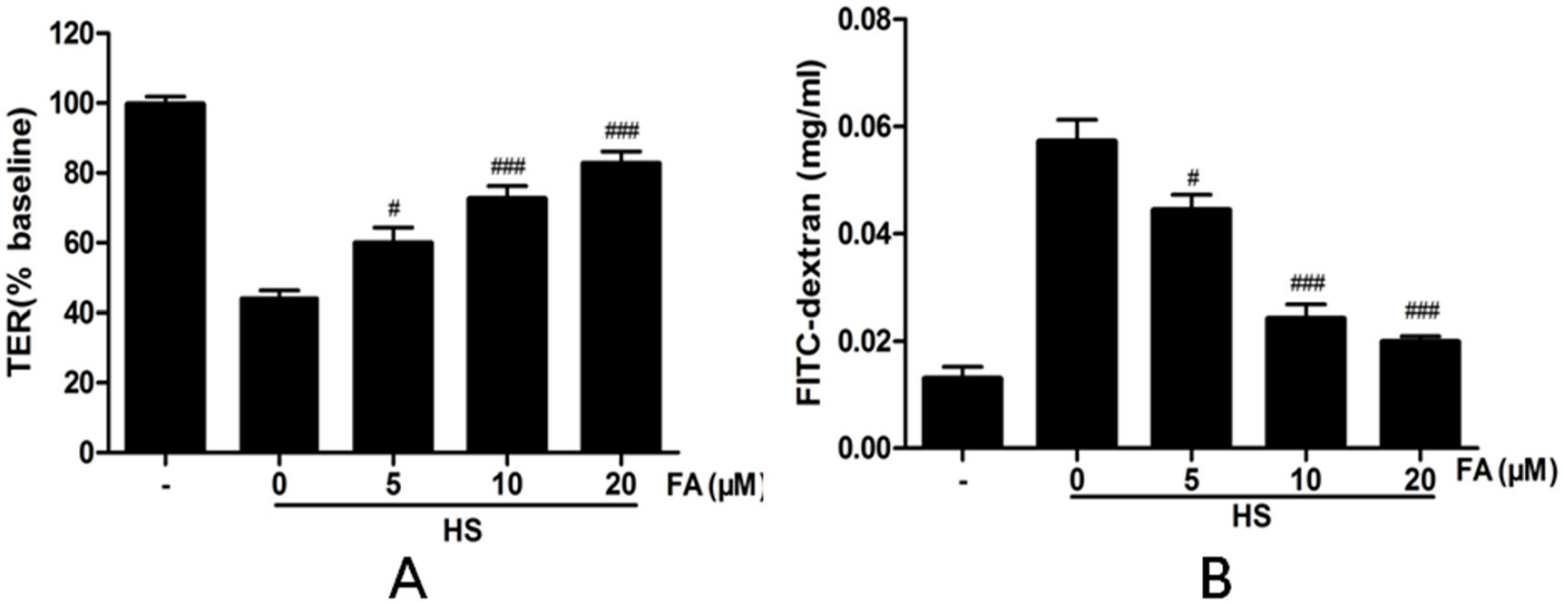
Effects of FA on heat stress-induced paracellular permeability in IEC-6 cell monolayers. Cells on transwell insert membranes were pretreated with FA (0, 5, 10 or 20 μM) for 4 h and then exposed to heat stress for 6 h. (A) TER values were monitored across the cell monolayers (B) Permeability of FD4 across the cell monolayer was measured. Data are presented as means ± SEM from three independent experiments and differences between mean values were assessed by one-way ANOVA. ^#^p<0.05, ^##^p< 0.01 and ^###^p<0.001 as compared with the heat stress group without FA.

Next, we investigated effects of FA on heat stress-induced changes in TER and FD4 flux. Heat stress significantly decreased TER in the IEC-6 monolayers, as compared with controls not subjected to heat stress. Pretreatment with FA (5, 10 and 20 μM) significantly attenuated this heat stress-induced TER decrease in a dose-dependent manner (Fig 4A). In addition, FA prevented the increase in paracellular permeability to FD4 induced by heat stress (Fig 4B).

### FA protected against heat stress-induced loss and redistribution of occludin, ZO-1 and E-cadherin proteins

**Fig 5:**
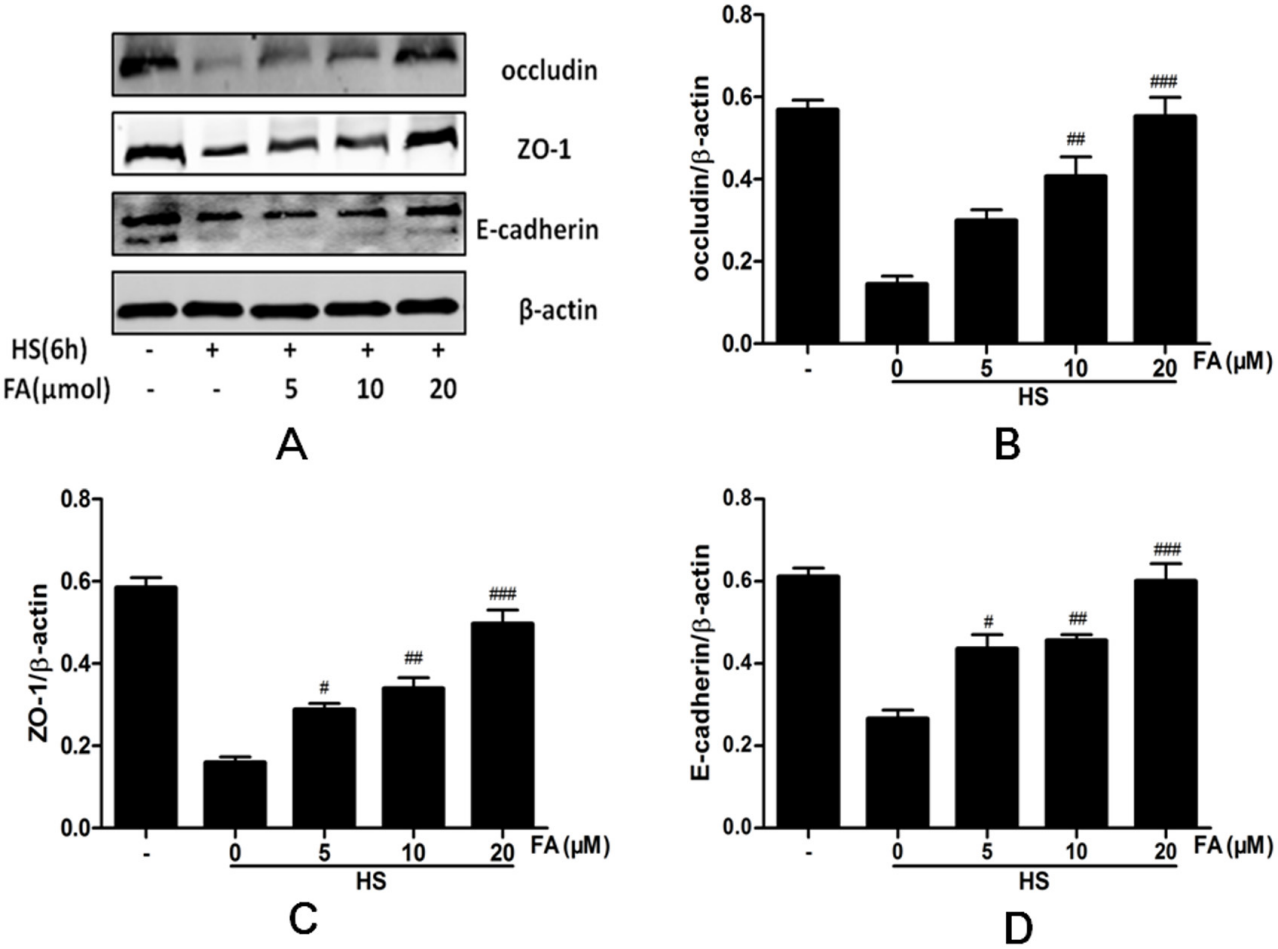
Effects of FA on heat stress-induced changes in protein expression in IEC-6 cells by western blot analysis. Cells were pretreated with FA (0, 5, 10 or 20 μM) for 4 h and then exposed to heat stress for 6 h. Immunoblot analysis was performed to detect the expression of the proteins occludin, ZO-1 and E-cadherin. Data are presented as means ± SEM from three independent experiments and differences between mean values were assessed by one-way ANOVA. ^#^p<0.05, ^##^p< 0.01 and ^###^p<0.001 as compared to heat stress group.

We investigated whether FA protected cells against the heat stress-induced loss of occludin, ZO-1 and E-cadherin. Heat stress significantly reduced expression of occludin, ZO-1 and E-cadherin proteins and FA prevented this down regulation in a dose-dependent manner (Fig 5).

**Fig 6:**
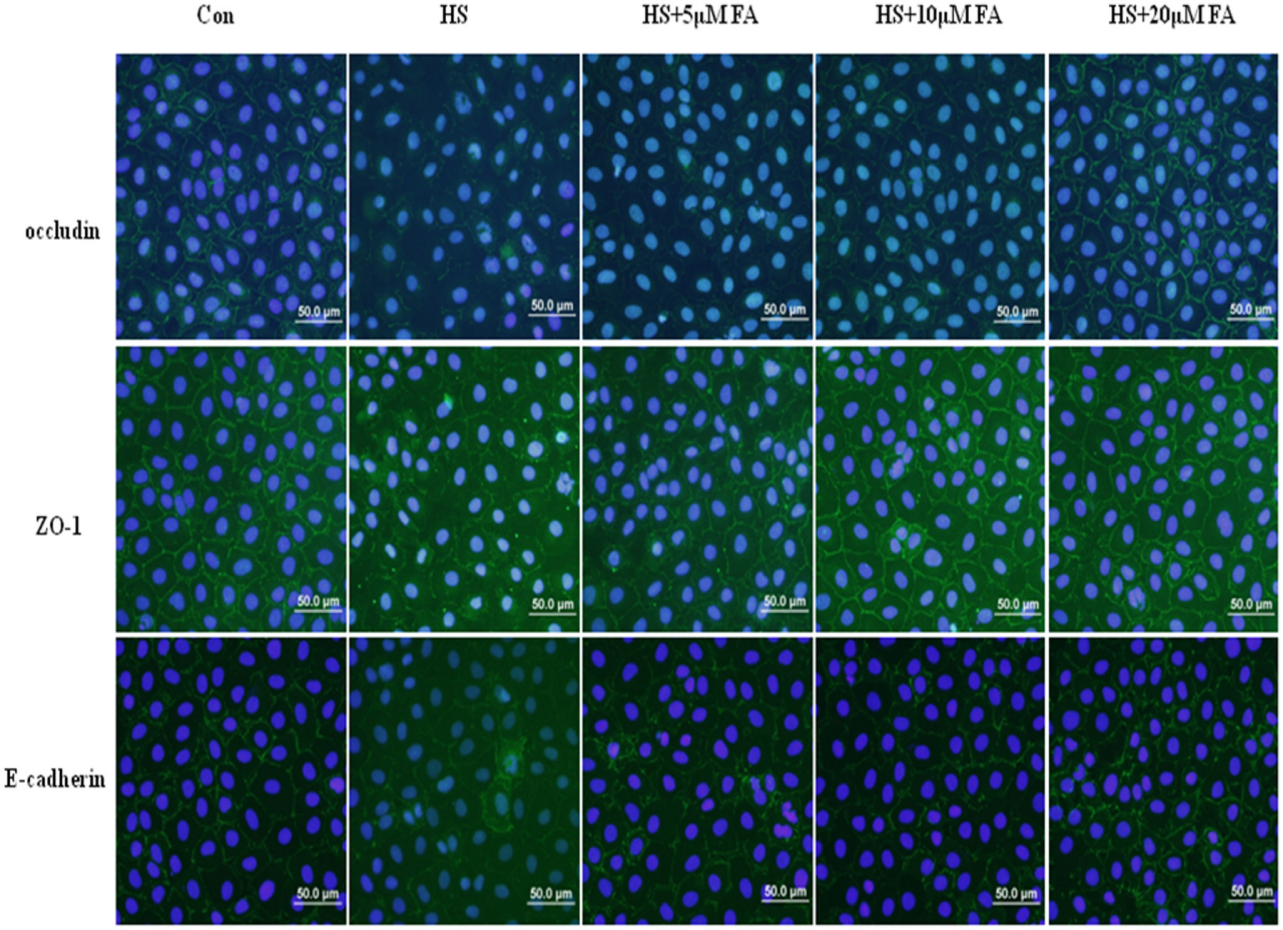
Effects of FA on junctional localization of occludin, ZO-1 and E-cadherin proteins by immunofluorescence. IEC-6 cells were pretreated with FA (0, 5, 10 or 20 μM) for 4 h and then exposed to heat stress for 6 h. The figure shows representative results from three independent experiments. Scale bar = 50 μm.

Impacts of FA on heat stress-induced effects on the junctional localization of the occludin, ZO-1 and E-cadherin proteins were assessed with immunofluorescence staining (Fig 6). In the control group not subjected to heat stress, occludin, ZO-1 and E-cadherin localized at the apical cellular junctions and appeared as a continuous band encircling the cells at the cellular borders. Heat exposure for 6 h caused a striking disruption to the localization of occludin, ZO-1 and E-cadherin proteins at the cellular borders. This was characterized by decreased staining intensity and marked cytoplasmic accumulation. In cells pretreated with 5 μM FA, intensities of staining for occludin, ZO-1 and E-cadherin proteins were slightly increased. These intensities were significantly increased with 10 μM FA treatment. Furthermore, in the group receiving 20 μM FA, localization and intensity of staining for occludin, ZO-1 and E-cadherin proteins were similar to staining in cells not subjected to heat stress.

### FA prevented heat stress-induced morphological disruption of the TJs

**Fig 7:**
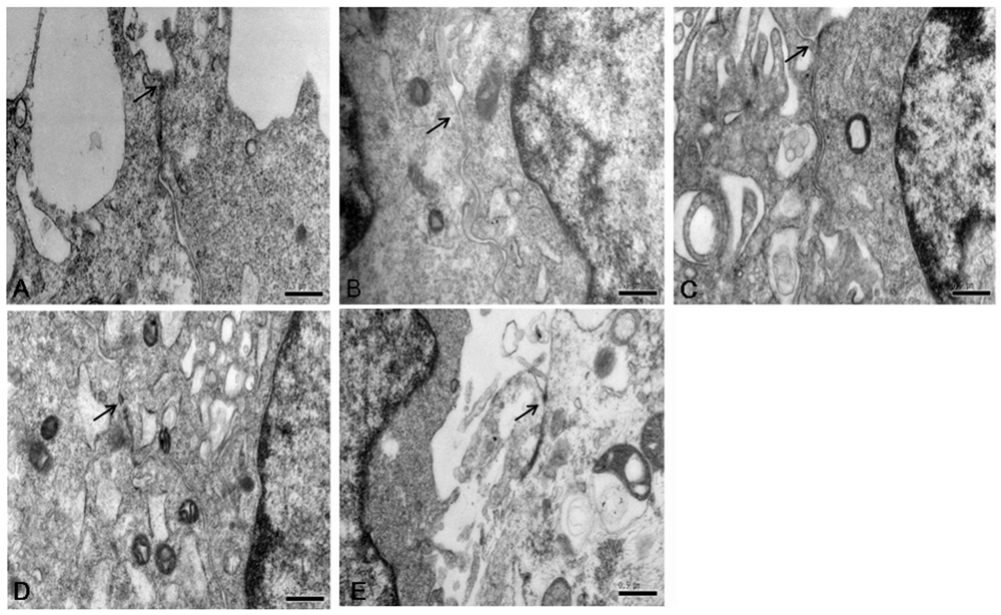
Effect of FA on morphological ultrastructure of tight junction induced by heat stress. Ultrastructure of TJs in IEC-6 monolayers cell was observed with a transmission electron microscope. (A) Control group. (B) Heat stress group. (C) 5 μM FA-pretreated heat stress group. (D) 10 μM FA-pretreated heat stress group. (E) 20 μM FA-pretreated heat stress group. Arrows indicate the location of the TJs (Scale bar = 0.5 μm).

Ultrastructure of IEC-6 cells was investigated by transmission electron microcopy (Fig 7). In the control group, TJs displayed intact structure between adjoining cells. After heat stress, TJs became markedly “open” with large gaps between adjoining cells and the electron-dense material of TJs was diminished. However, cells subjected to heat stress and also pretreated with 5 μM FA showed a slightly alleviated change in TJs morphology, and this improvement was significant with 10 μM FA. In cells pretreated with 20 μM FA, the TJs showed an intact ultrastructure, comparable to that of cells not subjected to heat stress.

### FA attenuated heat stress-induced increases in intestinal permeability *in vivo*

**Fig 8:**
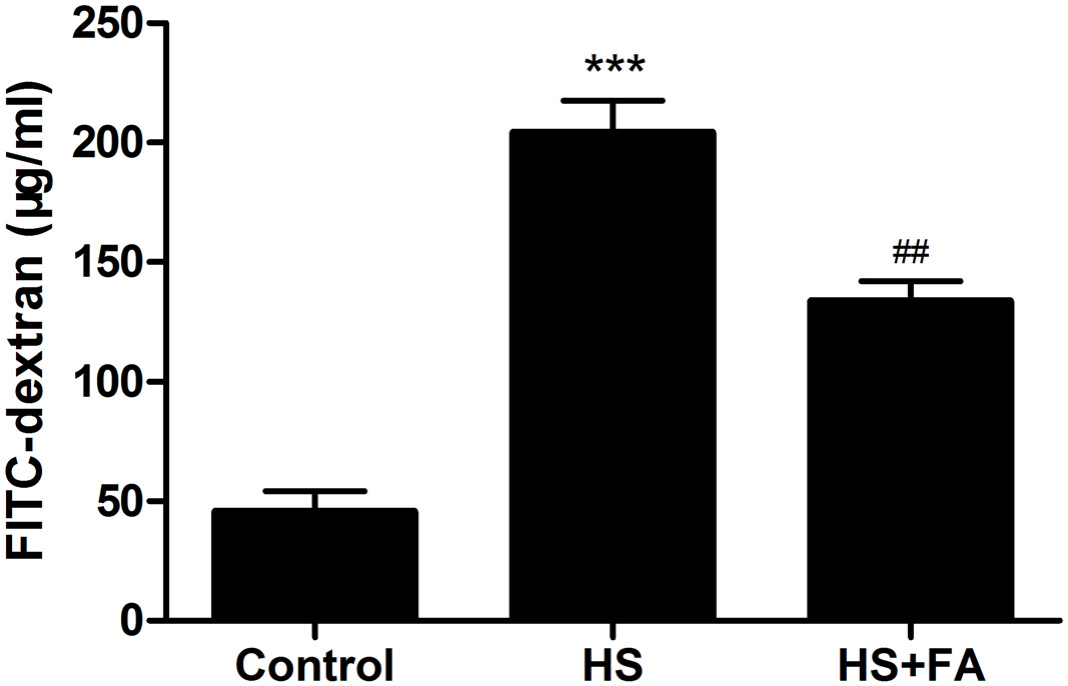
Effects of FA on intestinal permeability in heat stressed rats. Rats were pretreated with normal saline for the control and heat stress (HS) groups, or with FA before heat stress. Intestinal permeability was determined based on plasma FD4, measured by fluorescence spectrophotometry. Data are presented as means ± SEM from three independent experiments and differences between mean values were assessed by one-way ANOVA. *** p<0.001 compared with the control group and ^##^p< 0.01 as compared to the heat stress group without FA.

Intestinal permeability was evaluated *in vivo* after heat stress using FD4 (Fig 8). Rats in the heat stress group had significantly higher levels of plasma FD4, as compared with the control group. However, in rats also pretreated with FA, this heat stress-induced FD4 permeability increase was diminished.

### FA prevented heat stress-induced morphological disruption of the TJs *in vivo*

**Fig 9:**
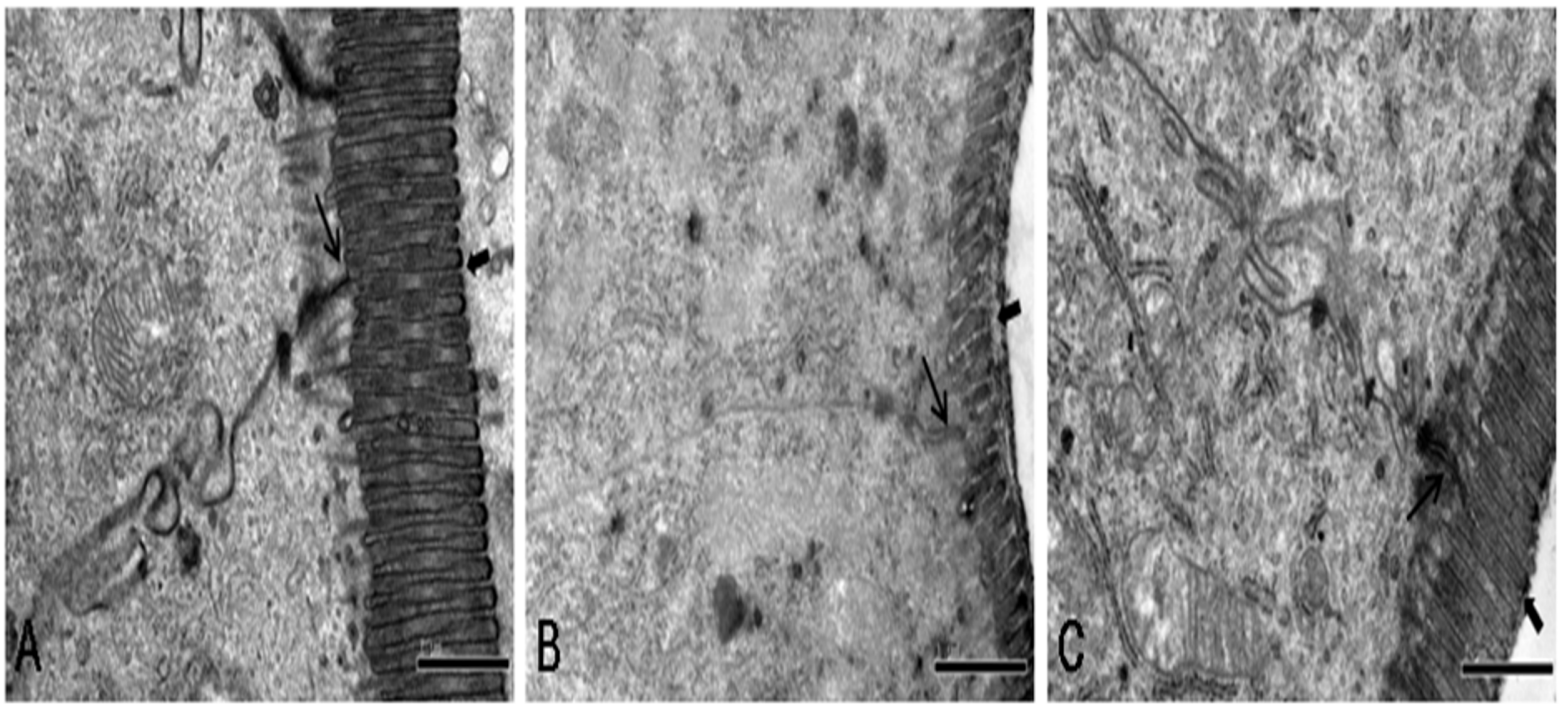
Effects of FA on morphological ultrastructural changes in TJs and microvilli induced by heat stress. Ultrastructure of TJs and microvilli in rat jejunum was observed by transmission electron microscopy. (A) Control group. Rats were pretreated with normal saline (B) Heat stress (HS) group. Rats were pretreated with normal saline and then exposed to heat stress (C) FA+ HS group. Rats were pretreated with FA and then exposed to heat stress. Thin arrows indicate TJs, and Thick arrows indicate microvilli. (Scale bar = 1 μm).

The ultrastructure of the jejunum was investigated by transmission electron microcopy (Fig 9). In the control group, TJs were intact between adjoining cells and the cells had regularly aligned microvilli. After heat stress, TJs between cells were lost and irregularly wide and the microvilli appeared damaged and were shorter. However, rats subjected to heat stress and also pretreated with FA had significantly decreased morphological changes in TJs and microvilli in response to heat stress compared with those not receiving FA.

### FA protected against heat stress-induced loss of occludin, ZO-1 and E-cadherin proteins

**Fig 10:**
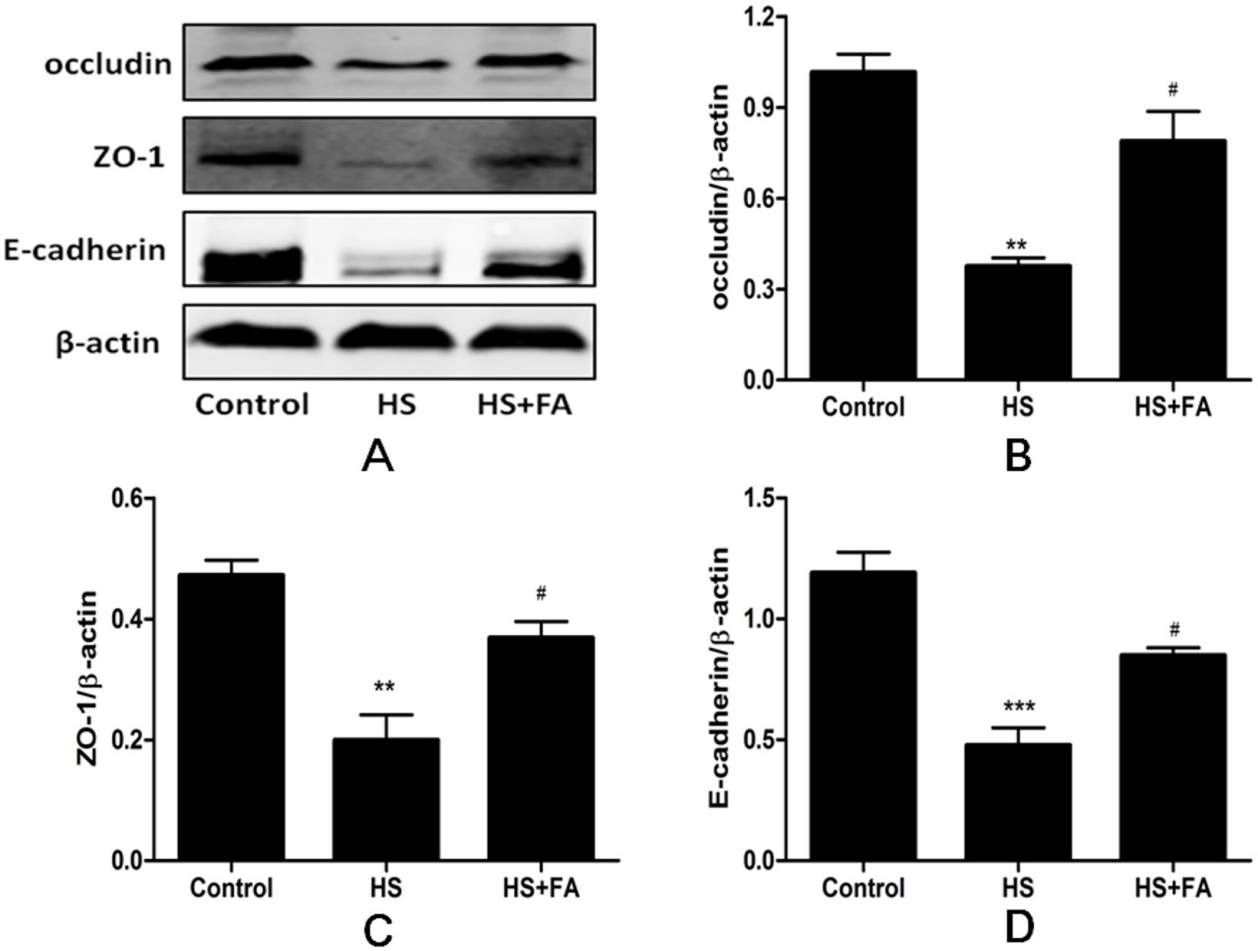
Effects of FA on intestinal occludin, ZO-1 and E-cadherin protein levels in heat stressed rats. Intestinal samples were collected after heat stress. Western blotting analysis was performed to detect levels of occludin, ZO-1 and E-cadherin proteins. Data are presented as means ± SEM from three independent experiments and differences among mean values were assessed by one-way ANOVA. ** p<0.01, *** p<0.001 compared with the control group, ^#^p< 0.05 as compared to the heat stress group without FA.

To further investigate the protective effect of FA against heat stress-induced intestinal epithelial barrier damage *in vivo*, levels of TJ proteins in the rat jejunum were analyzed by western blotting (Fig 10). Levels of occludin, ZO-1 and E-cadherin proteins were significantly lower after heat stress. However, in rats subjected to heat stress and pretreated with FA, occludin, ZO-1 and E-cadherin were significantly higher, compared with those receiving heat stress but no FA.

## Discussion

Functional defects in the intestinal epithelial barrier are important pathogenic factors in disorders such as celiac disease, inflammatory bowel disease, sepsis and cardiovascular disease [21,22]. Heat stress is regarded as being a contributor to intestinal epithelial barrier dysfunction by increasing intestinal permeability, thus enabling entry to pathogens and antigens [23]. Because of this, during heat stress, it is crucial to protect the function of intestinal epithelial barrier. In this study, we demonstrated that FA protects against heat stress-induced increases in intestinal epithelial permeability and the associated damage of epithelial junctional complexes.

The intestinal epithelium forms a selectively permeable barrier, and it is very important that the barrier between the gut lumen and internal tissues be maintained [21,22]. However, intestinal barrier dysfunction is considered as an important adverse effect of heat stress in Caco2 cell model [23,24]. Meanwhile, when mice exposed to heat stress, intestinal epithelial barrier permeability and plasma endotoxin concentrations and bacterial load in the blood, spleen and mesenteric lymph nodes were all increased [24]. The present study showed that heat stress reduced TER and increased permeability for the macromolecule FD4 across IEC-6 cell monolayers. These findings indicate that the intestinal epithelium barrier was physically impaired with heat stress. Our results also showed that the effects on TER and FD4 were greater with longer periods of heat stress. In IEC-6 cells exposed to heat stress for 6 h, TER was significantly reduced and FD4 flux significantly increased. Based on this, we selected a 6-h exposure to heat stress as the model condition for many experiments in our study.

The TJs and AJs control epithelial homeostasis, paracellular permeability and barrier properties [25]. TJ proteins include the transmembrane proteins occludin and claudins and the peripheral membrane proteins ZO-1 and ZO-2. E-cadherin is the major component of AJs, essential for intestinal development, homeostasis and the defense against pathogenic bacteria [26,27]. Because TJ and AJ proteins are involved in maintenance of epithelial barrier function, we investigated the role of TJ- and AJ-associated proteins, occludin, ZO-1 and E-cadherin, in the effects of heat stress in our model. These proteins are all directly involved in establishing and regulating intestinal barrier function [28,29]. Previous studies have shown that inhibition of occludin protein expression in intestinal epithelial cells led to increased paracellular permeability to macromolecules, indicating that occludin is critical to maintenance and assembly of the TJ barrier [30,31]. Heat stress (39 or 41°C) increased occludin protein expression and mRNA transcription in Caco-2 cells, which is mediated by HSF-1 activation and subsequent binding of HSF-1 to the occludin promoter [32]. However, in our study, occludin protein expression increased at 3 h of heat stress, but decreased dramatically, as compared to cells maintained at normal temperature, at 6 and 12 h of heat stress. ZO-1 acts as a multi-domain scaffolding protein and the coordinated activity of multiple conserved domains is required for TJ structure and maintenance of epithelial permeability [33]. Recent studies showed that heat stress decreased ZO-1 protein expression in Caco-2 cells [5,32]. Our results were consistent with this, showing that ZO-1 protein expression was significantly reduced in IEC-6 cells exposed to heat stress for 3 to 12 h. E-Cadherin is the major component of AJs, essential for intestinal development, homeostasis, and defense against pathogenic bacteria [26,27]. E-Cadherin facilitates assembly of specialized cellular junctions such as TJs and desmosomes, which together are required to connect epithelial cells into a functional monolayer [34–36]. Our findings showed that expression of E-cadherin was increased after 3 h of heat stress, but significantly decreased with continued heat stress, at 6 and 12 h time points.

FA is a ubiquitous phenolic compound in vegetables, fruits and some Chinese medicinal herbs [37]. It is a strong antioxidant, effectively scavenging free radicals and inhibiting lipid peroxidation [15,38]. Recent research demonstrated that ferulate prevented tert-butyl hydroperoxide-induced increases in paracellular permeability in Caco-2 cells [18]. Therefore, we investigated whether FA could protect heat stress-induced increases in paracellular permeability IEC-6 cells. In our study, results from the MTT assay showed that FA was not cytotoxic for IEC-6 cells at concentrations as high as 500 μM. This indicated that the effects on permeability that we observed with much lower FA concentrations (up to 20 μM) were not due to cytotoxicity. Our results also demonstrated that FA, in a dose-dependent manner, can prevent heat stress-induced decreases in TER and increases in FD4 flux. Therefore, our results indicate that FA effectively inhibits heat stress-induced increased paracellular permeability in IEC-6 cells.

Both decreased expression and altered localization of apical junctional proteins are related to epithelial barrier function disruption [39,40]. Our data showed that heat stress significantly downregulated occludin, ZO-1 and E-cadherin in intestinal epithelial cells and that this down regulation was attenuated, in a dose-dependent manner, by FA. By immunofluorescence, heat stress reduced staining intensity of occludin, ZO-1 and E-cadherin proteins at the cellular borders and induced translocation of these proteins from the membrane into the cytosol. However, pretreatment with FA markedly prevented this translocation of occludin, ZO-1 and E-cadherin proteins from the membrane of IEC-6 cells subjected to heat stress. Moreover, in heat stressed cells, we observed a morphological change of TJs by TEM. Heat stress induced disappearance of electron-dense material in TJs. FA pretreatment alleviated these morphological changes in a dose-dependent manner. These results indicate that FA can protect the epithelial barrier during heat stress by maintaining expression and stabilizing localization of apical junctional proteins.

Furthermore, we investigated protective effects of FA against heat stress-induced intestinal epithelial barrier damage in a rat model. Many studies have shown that heat stress increased intestinal permeability and damaged intestinal barrier function *in vivo* [19,41]. The results of our *in vivo* permeability assay also showed that heat stress significantly increased plasma FD4 levels and that this effect was significantly diminished by pretreatment with FA. Structurally intact TJs are important for establishing and regulating intestinal barrier function. In recent reports, heat stress-induced destruction of TJ structure was proposed to result in intestinal barrier dysfunction and increased permeability [5,42]. In our study, the TJs between cells were lost and irregularly wide and the microvilli were damaged and shortened in the heat stress group. Pretreatment with FA significantly decreased these heat stress-induced morphological disruptions to TJs and microvilli. Because the expression of TJ proteins directly influence TJ morphology, we also investigated expression of occludin, ZO-1 and E-cadherin proteins by western blotting. We found that levels of these TJ proteins were significantly reduced after heat stress. However, pretreatment with FA before heat stress led to significantly higher expression of occludin, ZO-1 and E-cadherin proteins compared with in the heat stress group. Therefore, these results indicate that pretreatment with FA alleviated heat stress-induced intestinal barrier disruption in the rat.

## Conclusion

Our study demonstrated for the first time that FA pretreatment was protective against heat stress-induced intestinal epithelial barrier dysfunction in vitro and in vivo. These findings provide evidence that pretreatment with FA may offer a therapeutic option for preventing heat stress-induced intestinal barrier dysfunction. Further investigations will be required to elucidate the precise mechanisms underlying these FA-mediated protective effects on intestinal epithelial barrier function.

## Supporting Information

S1 Table Relevant data underlying the findings described in manuscript. (PZF)
